# Taking the ‘I’ out of LLINs: using insecticides in vector control tools other than long-lasting nets to fight malaria

**DOI:** 10.1186/s12936-020-3151-x

**Published:** 2020-02-14

**Authors:** Krijn P. Paaijmans, Silvie Huijben

**Affiliations:** 1 Center for Evolution and Medicine, School of Life Sciences, Arizona State University, Tempe, AZ USA; 2 The Biodesign Center for Immunotherapy, Vaccines and Virotherapy, Arizona State University, Tempe, AZ USA; 3 ISGlobal, Barcelona, Spain; 4 Centro de Investigação em Saúde de Manhiça (CISM), Maputo, Mozambique

**Keywords:** Insecticides, Resistance, Malaria elimination, Vector control

## Abstract

Long-lasting insecticidal nets, or LLINs, have significantly reduced malaria morbidity and mortality over the past two decades. The net provides a physical barrier that decreases human-mosquito contact and the impregnated insecticide kills susceptible mosquito vectors upon contact and may repel them. However, the future of LLINs is threatened as resistance to pyrethroids is now widespread, the chemical arsenal for LLINs is very limited, time from discovery of next-generation insecticides to market is long, and persistent transmission is frequently caused by vector populations avoiding contact with LLINs. Here we ask the question whether, given these challenges, insecticides should be incorporated in nets at all. We argue that developing long-lasting nets without insecticide(s) can still reduce vector populations and provide both personal and community protection, if combined with other approaches or technologies. Taking the insecticide out of the equation (i) allows for a faster response to the current pyrethroid resistance crisis, (ii) avoids an LLIN-treadmill aimed at replacing failing bed nets due to insecticide resistance, and (iii) permits the utilization of our current and future insecticidal arsenal for other vector control tools to target persistent malaria transmission.

## Background

Insecticide-treated bed nets (ITNs), later replaced by long-lasting insecticidal nets (LLINs), have undoubtedly played a major role in reducing malaria cases since 2000. Sixty-eight percent of the overall malaria reductions observed between 2000 and 2015 may be attributed to nets [1]. Two main factors contribute to their success story: First, the properties of the LLIN allow for a very effective intervention tool: it targets indoor-biting mosquitoes (arguably responsible for the bulk of malaria cases historically), is highly effective as a physical barrier (reducing human-vector contact) and contains an insecticide that kills susceptible mosquitoes upon or after contact with the net. In addition, pyrethroid insecticides, the only chemical class currently used in all nets, can have an excito-repellent effect, diverting mosquitoes before they feed. Second, the large-scale mass LLIN distribution has led to astonishing numbers of LLINs being deployed, mainly in sub-Saharan Africa. To illustrate, between 2010 and mid-2019 over 1.70 billion pyrethroid-based bed nets have been distributed, of which 1.43 billion in sub-Saharan Africa [2]. Given their success, LLINs remain a core intervention for National Malaria Control Programmes. Pillar 1 of the Global Technical Strategy for Malaria 2016–2030 envisions universal coverage for all people at risk of malaria using effective vector control with either LLINs (i.e. one net for every two persons at risk of malaria [3]) or indoor residual spraying (IRS), the other core prevention tool [4].

## The threats of insecticide resistance and the global response

The flipside of the impressive rollout of exclusively pyrethroid-based bed nets is the rapid spread of pyrethroid resistance throughout sub-Saharan Africa [5, 6], which has been exacerbated by the concomitant use of the same chemical class in IRS campaigns [7]. The programmatic impact of pyrethroid resistance is not well understood and topic of considerable debate. A recent multi-country observational cohort study showed no association between insecticide resistance on malaria transmission [8], yet another recent study showed that malaria infection prevalence was significantly reduced in areas that received pyrethroid-based LLINs containing a synergist (piperonyl-butoxide or PBO, which enhanced the efficacy of the pyrethroid insecticides against resistant mosquitoes), hinting that insecticide resistance may indeed have a programmatic impact [9].

The key question is how to respond to the insecticide resistance crisis? The current global response is to develop novel insecticides as well as resistance management strategies. Resistance management strategies that aim to slow down the emergence and spread of insecticide resistance include (i) avoidance of the use of pyrethroids for IRS when pyrethroid-LLINs are present in same area [10], and (ii) the need to rotate or mix insecticides or apply mosaics (applicable to IRS, but could arguably be used in future LLIN delivery strategies as well) as highlighted by the global plan of the World Health Organization (WHO) for insecticide resistance management (GPIRM) [11]. The latter has not been operationally deployed at scale, due to the lack of robust, timely and accurate insecticide resistance surveillance, and the lack of coordination between entomological surveillance and procurement practices, amongst other reasons. Although there is little evidence from the field on which of the strategies is best [12], increased support through mathematical modelling [see e.g. 13] is expected, as field trials will be too expensive and time-consuming. Combining modelling with the collection of empirical data may allow us to explore how different insecticide use strategies will affect resistance evolution [13].

In addition to managing resistance to the insecticides currently available, there has been an increased investment in the development of new active ingredients (not necessarily to be used for LLINs). The new generation of LLINs combine a pyrethroid with a partner chemical. Pyrethroid-LLINs with the synergist PBO (see above) or chlorfenapyr (dual chemistry or mixture approach) have been pre-qualified by the WHO [14]. Novel chemistries are expected in two waves (but note these will not be dedicated to bed nets per se). The first wave is expected after 2022, when insecticides from three novel compounds, with no cross-resistance to current insecticide classes, will become available [5]. After that, new active ingredients generated under the ZeroX40 initiative (an initiative of five chemical companies with the support of the Bill & Melinda Gates Foundation and the Innovative Vector Control Consortium) are expected to become available. However, as time from discovery to WHO prequalification (often a prerequisite for donors to consider a vector control product) can easily be 10 years [15], the vector control arena will have to wait a considerable amount of time for new classes of insecticides to reach the market.

## Where to (not) deploy our chemical arsenal?

It appears the malaria elimination community has entered the “insecticide treadmill”, meaning we aim to create an open-ended development pipeline to continuously target resistant mosquitoes with new chemistries. Given that (i) there is a very limited availability of effective public health insecticides now and in the near future [5, 6], and (ii) we will be facing challenges in increasing domestic and international funding [16], insecticide-use practices should be carefully evaluated as they exert pressure on the useful lifespan of the chemical and thus on all its associated intervention tools. Here we focus on the major front-line vector control tool, the LLIN, and propose to transform them into long-lasting nets without the insecticide(s). We will refer to such nets as LLUNs (long-lasting untreated nets). We purposely have not removed long-lasting (LL): Although this term is associated with the insecticide, net durability is key to LLUN success, as we will explained below.

Apart from the rationale above, there are additional arguments to consider (such as comfort, health risks and environmental impact, see Box 1), but one clearly stands out for us: LLINs (and IRS) have led to (i) other vector species (known secondary vectors, previously undescribed vectors as well as species not thought to be vectors [17]) becoming important, which often have different feeding behaviours (outdoor feeding and/or preference for animal feeding) [18, 19] and/or (ii) changes in biting behaviours (to outdoors or different times indoors) in the historically dominant vectors [20, 21]. These behaviours are not specifically targeted by LLINs, but can by a few other interventions. Considering the physical barrier of the bed net, we ask here whether our limited chemical arsenal could be more impactful—in terms of lives saved—when applied to other interventions that may intrinsically depend on insecticides for functionality. Examples include window screens, wall liners, attractive targeted sugar baits, eave tubes, and outdoor barrier screens amongst others [see e.g. 22].

**Fig. 1:**
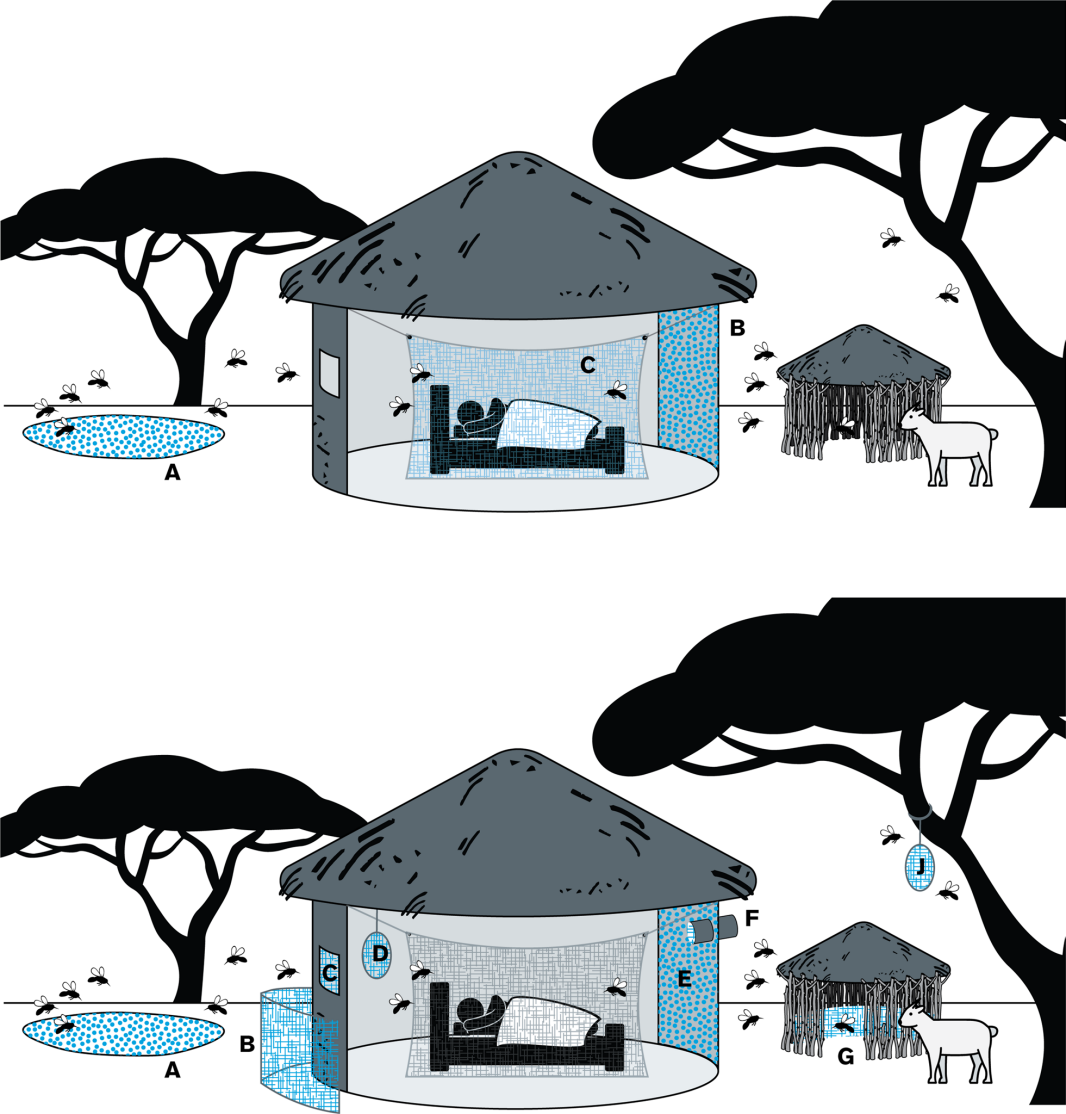
Top panel: Areas that are currently being targeted with insecticides, which include breeding sites (larvicides **a**) and inside houses (adulticides on walls **b** and/or in bed nets **c**). Bottom panel: Tools that may require insecticides in the future to have an impact. These include, but are not limited to, larvicides (**a**), barrier screens (**b**), window/eave screening (**c**), attractive targeted sugar baits (**d**, **j**), IRS (**e**, **g**), eave tubes (**f**), and/or wall liners (**e**, **g**)

Using the limited chemical arsenal for such tools (Fig. 1) may eventually provide more impactful vector control in the face of mounting resistance and the need to target different mosquito behaviours. A few things are worth noting: First, the epidemiological impact of most of these other vector control tools has not been properly assessed through Phase III vector control field studies [23]. Second, some of these interventions could also function without insecticides (window screens for example, see [24]). Third, pesticides not approved for the use in human health may be suitable for some of the interventions (e.g. attractive targeted sugar baits and eave tubes) as pesticides may be out of reach for people (assuming these will not aerosolize), whereas insecticide choice will be driven by public health concerns for interventions such as IRS, wall liners, window screens and arguably barrier screens if placed in close proximity to people. Finally, using insecticides elsewhere could similarly lead to the evolution of insecticide resistance if resistance is not managed properly [11, 25]. However, thinking about where and when to use our insecticides will broaden the number of effective tools available and extend the useful lifespan of the available tools.

## Could insecticide-free nets still protect the community?

The success of LLINs in reducing the global malaria burden has been attributed to the community protection effect: If net use exceeds a certain threshold (around 60% may already be sufficient [26]), overall mosquito densities and their survival are (in an insecticide-susceptible population) impacted sufficiently to also protect those individuals not using a net. Whilst an LLUN would still provide personal protection, by way of the physical barrier that reduces vector-human contact, the community protection may be lost, as vectors are not killed upon contact and mosquito densities not reduced. However, the causality of the community effect is not empirically established nor understood. Increasing mosquito mortality and reducing mosquito biting rate both reduce the basic reproductive number as can be derived from the Ross-Macdonald model [27]. To what extent local transmission intensity is lowered by the killing efficacy of an LLIN or by the reduction of mosquito-host contact through the physical barrier has not been studied. The quantitative loss of community effect by not introducing an insecticide in an insecticide susceptible population is not presently known, and in the face of the current widespread insecticide resistance this loss may even be negligible. Moreover, evidence suggest that hosts under an LLIN could be more attractive to resistant mosquitoes (*kdr* homozygous) than those sleeping under an untreated net [28]. A randomized controlled trial could tease out the role of the barrier *versus* the role of the insecticide(s). Although this may be considered unethical, and it arguably was when LLINs hit the market and mosquitoes were susceptible to the insecticides used in them, the nature of this argument has since changed considerably.

This discussion aside, long-lasting untreated nets (LLUNs) could certainly preserve a level of community protection similar to effective LLINs through some simple modifications in design and/or by combining them with other vector control tools to reduce overall mosquito densities. For instance, LLUNs could act as a non-chemical ‘human-baited trap’. Mosquitoes are known to approach both LLINs and untreated nets at the top [29, 30]. By designing an LLUN-trap at the top of the net, mosquitoes can ‘enter’ the bed net (into e.g. a separate compartment such as a bottle) but not leave. Alternatively, a patch could be placed on top of the net with e.g. a sticky layer to catch mosquitoes (similar to the SmartPatch [31] but with a non-insecticidal solution). Patches could be coated with chemicals that not directly aimed at killing the adults upon contact, but target e.g. mosquito progeny via auto-disseminating insecticides that are carried by adult mosquitoes to breeding sites, killing their and the progeny of others [32]. In addition, a repellent may be added to strengthen push–pull strategies [33]: As LLUNs attract host-seeking vectors looking to bite the person(s) sleeping under the net, they may be ‘pushed’ to another device, the ‘pull’ (e.g. an attractant lethal trap [34]), after they are unsuccessful in obtaining their blood meal.

These is a non-exhaustive list of suggestions that could allow LLUNs to remove mosquitoes from the overall vector population, while providing a similar community protective effect. This approach would preserve the useful lifespan of our limited arsenal of insecticides to other, insecticide-dependent, interventions. The feasibility of such alternative approaches to LLINs should be evaluated by an interdisciplinary community of industry, mosquito biologists and behaviourist, evolutionary biologists, engineers, social scientists, health economists and modelers who work together to develop prototype LLUN designs that will meet intervention goals, acknowledging there may be areas where LLINs will remain the most useful form of protection.

## Conclusions

The malaria elimination community has now entered the “insecticide treadmill” to keep up with the emergence and spread of insecticide-resistant malaria vectors. But given that (i) the current insecticide toolbox is limited, (ii) the wait for additional new chemistries will be long, and (iii) shifts in mosquito behavioural traits have been observed in many locations as a result of our interventions, we need to think about the most cost-effective and most sustainable strategies for our current, and in particular, novel insecticides [25]. We argue that an evidence-based discussion is urgently needed before the next generation of insecticidal vector control tools enters the development pipeline. Are insecticides always the critical component to make an intervention effective or can alternative strategies be engineered? We believe that developing long-lasting nets without insecticide(s) can still reduce vector populations and provide both personal and community protection, if combined with other approaches or technologies. Doing so would protect an insecticide from resistance selection and thus allows the insecticide to be used in other interventions. However, it would require a paradigm shift in the malaria control community.

Box 1. Additional benefits of LLUNs over LLINs

- Cost
  Not using insecticides eliminates the chemical procurement costs and the costs associated with the impregnation of the insecticide(s) into the bed net. Arguably, the gains should be invested in LLUN improvement (as explained in the main text) and in the areas highlighted below (b-d).
- Durability
  LLINs integrity [35, 36] is not always on par with the 3 years replacement recommendations set forward by the WHO [3]. In the absence of chemical lifespan and associated material restrictions, manufacturers could focus on improved net durability, maximizing its non-chemical protection (e.g. stronger and/or self-repairing materials, inclusion of repair sets).
- Increased protective effect
  As LLUNs will not have a repellent effect as some LLINs, an additional/different layer of protection may be needed around those body parts that are easily exposed to mosquitoes (when the LLUN is in contact with e.g. the arms, legs or feet) by e.g. adding a strip of impenetrable fabric, which reduces the likelihood of mosquito bites and hence probability of transmission.
- Comfort
  Bed nets, treated or untreated, are often not used due to discomfort, primarily due to heat [37]. Nets can be redesigned to improve airflow, and the addition of e.g. a fan [38] and/or an LED light may increase LLUN usage.
- Reduced health risks
  Although the insecticides that are incorporated in nets are WHO-approved, they could—in theory—lead to longer-term health risks that are not identified during the product testing phases [39]. It is worth noting that LLIN users are in close contact with the net throughout the time they use it.
- Environmental impact
  The amount of polymer in one LLIN translates to roughly 500 g of plastic, which is equivalent 40–50 plastic bags [40]. With the current LLIN replacement policy of 3 years [3], the 100s of millions of LLINs distributed annually [2] and the fact that LLIN collection is not implemented on a large scale [41], large amounts of plastic will eventually end up in the environment. Worryingly, a certain dose of pesticide will remain in those nets for several years after disposal [40]. Although a cohort of nets will undoubtedly be repurposed, we were unable to find quantifiable evidence.

## Data Availability

Not applicable.

## Abbreviations

GPIRM: Global plan for insecticide resistance management
IRS: Indoor residual spraying
ITN: Insecticide-treated net
LLIN: Long-lasting insecticidal nets
LLUN: Long-lasting untreated nets
PBO: Piperonyl-butoxide
WHO: World Health Organization

## References

[1] Bhatt S, Weiss DJ, Cameron E, Bisanzio D, Mappin B, Dalrymple U (2015). The effect of malaria control on *Plasmodium falciparum* in Africa between 2000 and 2015. Nature.

[2] Roll Back Malaria. The Alliance for Malaria Prevention. Net Mapping 2nd Q 2019. https://allianceformalariaprevention.com/wp-content/uploads/2019/07/AMP-Net-Mapping-2nd-Q-2019.xlsx. Accessed 19/09/2019.

[3] WHO (2017). Achieving and maintaining universal coverage with long-lasting insecticidal nets for malaria control.

[4] WHO (2015). Global technical strategy for malaria 2016-2030.

[5] Hemingway J, Shretta R, Wells TNC, Bell D, Djimdé AA, Achee N (2016). Tools and strategies for malaria control and elimination: What do we need to achieve a grand convergence in malaria?. PLoS Biol.

[6] Ranson H, Lissenden N (2016). Insecticide resistance in African anopheles mosquitoes: a worsening situation that needs urgent action to maintain malaria control. Trends Parasitol..

[7] Oxborough RM (2016). Trends in US President’s Malaria Initiative-funded indoor residual spray coverage and insecticide choice in sub-Saharan Africa (2008–2015): urgent need for affordable, long-lasting insecticides. Malar J..

[8] Kleinschmidt I, Bradley J, Knox TB, Mnzava AP, Kafy HT, Mbogo C (2018). Implications of insecticide resistance for malaria vector control with long-lasting insecticidal nets: a WHO-coordinated, prospective, international, observational cohort study. Lancet Infect Dis..

[9] Protopopoff N, Mosha JF, Lukole E, Charlwood JD, Wright A, Mwalimu CD (2018). Effectiveness of a long-lasting piperonyl butoxide-treated insecticidal net and indoor residual spray interventions, separately and together, against malaria transmitted by pyrethroid-resistant mosquitoes: a cluster, randomised controlled, two-by-two factorial design trial. Lancet.

[10] WHO (2014). Guidance for countries on combining indoor residual spraying and long-lasting insecticidal nets.

[11] WHO (2012). Global plan for insecticide resistance management in malaria vectors.

[12] Hemingway J, Penilla RP, Rodriguez AD, James BM, Edge W, Rogers H (1997). Resistance management strategies in malaria vector mosquito control. A large-scale field trial in Southern Mexico. Pestic Sci..

[13] South A, Hastings IM (2018). Insecticide resistance evolution with mixtures and sequences: a model-based explanation. Malar J..

[14] WHO (2019). List of WHO prequalified vector control products (11/4/2019).

[15] Hemingway J (2017). The way forward for vector control. Science.

[16] Patouillard E, Griffin J, Bhatt S, Ghani A, Cibulskis R (2017). Global investment targets for malaria control and elimination between 2016 and 2030. BMJ Glob Health..

[17] Lobo NF, Laurent BS, Sikaala CH, Hamainza B, Chanda J, Chinula D (2015). Unexpected diversity of *Anopheles* species in Eastern Zambia: implications for evaluating vector behavior and interventions using molecular tools. Sci Rep..

[18] Kiware SS, Chitnis N, Moore SJ, Devine GJ, Majambere S, Merrill S (2012). Simplified models of vector control impact upon malaria transmission by zoophagic mosquitoes. PLoS ONE.

[19] Cooke MK, Kahindi SC, Oriango RM, Owaga C, Ayoma E, Mabuka D (2015). ‘A bite before bed’: exposure to malaria vectors outside the times of net use in the highlands of western Kenya. Malar J..

[20] Sougoufara S, Diédhiou SM, Doucouré S, Diagne N, Sembène PM, Harry M (2014). Biting by *Anopheles funestus* in broad daylight after use of long-lasting insecticidal nets: a new challenge to malaria elimination. Malar J..

[21] Moiroux N, Gomez MB, Pennetier C, Elanga E, Djenontin A, Chandre F (2012). Changes in Anopheles funestus biting behavior following universal coverage of long-lasting insecticidal nets in Benin. J Infect Dis..

[22] Killeen GF, Tatarsky A, Diabate A, Chaccour CJ, Marshall JM, Okumu FO (2017). Developing an expanded vector control toolbox for malaria elimination. BMJ Glob Health..

[23] Wilson AL, Boelaert M, Kleinschmidt I, Pinder M, Scott TW, Tusting LS (2015). Evidence-based vector control? Improving the quality of vector control trials. Trends Parasitol..

[24] Killeen GF, Govella NJ, Mlacha YP, Chaki PP (2019). Suppression of malaria vector densities and human infection prevalence associated with scale-up of mosquito-proofed housing in Dar es Salaam, Tanzania: re-analysis of an observational series of parasitological and entomological surveys. Lancet Plan Health.

[25] Huijben S, Paaijmans KP (2018). Putting evolution in elimination: winning our ongoing battle with evolving malaria mosquitoes and parasites. Evol Appl.

[26] Killeen GF, Smith TA, Ferguson HM, Mshinda H, Abdulla S, Lengeler C (2007). Preventing childhood malaria in Africa by protecting adults from mosquitoes with insecticide-treated nets. PLoS Med..

[27] Mandal S, Sarkar RR, Sinha S (2011). Mathematical models of malaria—a review. Malar J..

[28] Porciani A, Diop M, Moiroux N, Kadoke-Lambi T, Cohuet A, Chandre F (2017). Influence of pyrethroïd-treated bed net on host seeking behavior of *Anopheles gambiae* s.s. carrying the kdr allele. PLoS ONE..

[29] Parker JEA, Angarita-Jaimes N, Abe M, Towers CE, Towers D, McCall PJ (2015). Infrared video tracking of *Anopheles gambiae* at insecticide-treated bed nets reveals rapid decisive impact after brief localised net contact. Sci Rep..

[30] Sutcliffe JF, Yin SJMJ (2014). Behavioural responses of females of two anopheline mosquito species to human-occupied, insecticide-treated and untreated bed nets. Malar J..

[31] MacDonald M (2015). Landscape of new vector control products.

[32] Lwetoijera D, Harris C, Kiware S, Dongus S, Devine GJ, McCall PJ (2014). Effective autodissemination of pyriproxyfen to breeding sites by the exophilic malaria vector *Anopheles arabiensis* in semi-field settings in Tanzania. Malar J..

[33] Menger DJ, Otieno B, de Rijk M, Mukabana WR, van Loon JJA, Takken W (2014). A push-pull system to reduce house entry of malaria mosquitoes. Malar J..

[34] Menger DJ, Omusula P, Wouters K, Oketch C, Carreira AS, Durka M (2016). Eave screening and push-pull tactics to reduce house entry by vectors of malaria. Am J Trop Med Hyg.

[35] Gnanguenon V, Azondekon R, Oke-Agbo F, Beach R, Akogbeto M (2014). Durability assessment results suggest a serviceable life of two, rather than three, years for the current long-lasting insecticidal (mosquito) net (LLIN) intervention in Benin. BMC Infect Dis.

[36] Githinji S, Herbst S, Kistemann T, Noor AM (2010). Mosquito nets in a rural area of Western Kenya: ownership, use and quality. Malar J..

[37] Pulford J, Hetzel MW, Bryant M, Siba PM, Mueller I (2011). Reported reasons for not using a mosquito net when one is available: a review of the published literature. Malar J..

[38] Jaeger MS, Briët OJT, Keating J, Ahorlu CK, Yukich JO, Oppong S (2016). Perceptions on the effect of small electric fans on comfort inside bed nets in southern Ghana: a qualitative study. Malar J..

[39] Viel J-F, Warembourg C, Le Maner-Idrissi G, Lacroix A, Limon G, Rouget F (2015). Pyrethroid insecticide exposure and cognitive developmental disabilities in children: The PELAGIE mother–child cohort. Environ Int.

[40] Ramanantsoa A, Wilson-Barthes M, Rahenintsoa R, Hoibak S, Ranaivoharimina H, Rahelimalala MD (2017). Can the collection of expired long-lasting insecticidal nets reduce their coverage and use? Sociocultural aspects related to LLIN life cycle management and use in four districts in Madagascar. Malar J..

[41] WHO (2014). Recommendations on the sound management of old long lasting insecticidal nets.

